# Extracorporeal shockwave therapy for zoster-associated pain: A retrospective analysis based on spinal musculoskeletal imbalance

**DOI:** 10.1097/MD.0000000000048410

**Published:** 2026-04-17

**Authors:** Fang Cheng, Yanzhong Yu, Jing Zhang

**Affiliations:** a Pain Clinic, The Affiliated Hospital of Hebei University of Engineering, Handan City, Hebei Province, China; b Dermatology Department, The Affiliated Hospital of Hebei University of Engineering, Handan City, Hebei Province, China.

**Keywords:** herpes zoster, pain management, postherpetic neuralgia, retrospective study, shockwave therapy, spinal musculoskeletal homeostasis

## Abstract

Herpes zoster-associated pain (ZAP) is a common sequela of varicella-zoster virus reactivation, particularly among older adults. Emerging evidence suggests that spinal musculoskeletal homeostasis (SMH) imbalance may contribute to localized degenerative changes associated with ZAP. Although extracorporeal shockwave therapy (ESWT) has shown promise in musculoskeletal rehabilitation, its efficacy in treating ZAP from the perspective of SMH remains underexplored. This retrospective study aims to evaluate the effectiveness of ESWT in ZAP patients with musculoskeletal imbalance as assessed by imaging-based SMH scores. This retrospective study reviewed clinical records of 82 hospitalized patients diagnosed with spinal-segmental ZAP between May 2023 and June 2024. Based on their treatment protocols, patients were divided into 2 groups: those receiving conventional treatment (C group, n = 41) and those receiving ESWT in addition to conventional therapy (E group, n = 41). Pain scores (Numerical Rating Scale, NRS), sleep quality (Pittsburgh Sleep Quality Index, PSQI), and Spinal Musculoskeletal Imaging Score (SMIS) were evaluated at 4 time points: 48 hours post-admission, after 1 week of hospitalization, and at 1 and 3 months post-discharge. Statistical analysis was performed using *t* tests and chi-square tests, with significance set at *P* < .05. Both groups had comparable baseline characteristics. After 1 week of treatment, both groups showed improvement in pain and sleep quality, but the ESWT group demonstrated a significantly greater improvement in PSQI (*P* < .05). At 1 month post-discharge, NRS, PSQI, and SMIS scores were significantly better in the ESWT group (*P* < .05). By 3 months, these differences further increased, with statistically significant reductions in all 3 indicators in the ESWT group (*P* < .01). The incidence of postherpetic neuralgia (PHN) was lower in the ESWT group (2 cases vs 13 cases in the control group). Retrospective analysis suggests that ESWT may be an effective adjunctive therapy for ZAP patients with spinal musculoskeletal imbalance, significantly improving pain, sleep quality, and spinal musculoskeletal structure, while potentially reducing the risk of PHN. These findings support the integration of ESWT into comprehensive ZAP management strategies based on musculoskeletal health assessments. Further prospective studies are needed to validate these results.

## 1. Introduction

Herpes zoster-associated pain (ZAP) is a significant public health concern, especially among older adults and immunocompromised populations. It arises from the reactivation of latent varicella-zoster virus in dorsal root ganglia or cranial nerve sensory ganglia, resulting in acute neuralgia along the affected dermatome.^[1-3]^ In many cases, ZAP persists beyond the acute phase, developing into postherpetic neuralgia (PHN), which is defined by pain lasting more than 3 months after rash resolution. PHN is notoriously resistant to standard treatment and is associated with long-term physical and psychological distress, sleep disturbance, and reduced quality of life.^[4-6]^ Despite advances in antiviral therapy, nerve block techniques, and pharmacologic interventions such as gabapentinoids and tricyclic antidepressants, a considerable number of patients continue to experience severe, intractable pain. This has prompted the exploration of alternative, non-pharmacological approaches to improve outcomes and prevent the chronicity of pain.

Emerging clinical and imaging evidence suggests that spinal musculoskeletal homeostasis (SMH) plays a critical role in the pathophysiology of ZAP. SMH refers to the structural and functional balance within the spinal musculoskeletal system, encompassing vertebral alignment, paraspinal muscle integrity, fascial elasticity, and biomechanical load distribution. Disruptions in this homeostatic balance – such as muscle atrophy, fatty infiltration, fascial inflammation, or vertebral degeneration – may reduce the resilience of spinal nerve roots to viral reactivation and impair immune defense mechanisms.^[7,8]^ This concept has led to a growing interest in evaluating musculoskeletal imaging patterns and biomechanical risk factors in ZAP patients. Extracorporeal shockwave therapy (ESWT) has emerged as a promising noninvasive modality widely used in orthopedics and rehabilitation medicine. It delivers acoustic pulses to targeted tissues, promoting neovascularization, anti-inflammatory effects, tissue remodeling, and pain modulation. Recent studies have extended its indications beyond tendinopathies and calcific lesions to include neuropathic pain and myofascial syndromes. However, there is a paucity of studies investigating the utility of ESWT in the context of ZAP, particularly in patients with documented musculoskeletal structural abnormalities.^[9-12]^

This study aims to bridge this gap by retrospectively evaluating the clinical efficacy of ESWT in hospitalized patients with spinal-segmental ZAP who exhibit SMH dysfunction based on musculoskeletal imaging and health assessments. By comparing pain severity (Numerical Rating Scale, NRS), sleep quality (Pittsburgh Sleep Quality Index, PSQI), and Spinal Musculoskeletal Imaging Score (SMIS) between patients receiving conventional therapy and those receiving adjunctive ESWT, this study seeks to provide a comprehensive analysis of treatment outcomes. Additionally, we assess the incidence of PHN in both groups to explore the potential long-term protective role of ESWT. Through the lens of spinal musculoskeletal homeostasis, this study highlights the importance of integrative, system-level approaches in managing ZAP and preventing chronic pain progression. The findings aim to support the incorporation of musculoskeletal-based interventions into multidisciplinary ZAP treatment protocols and provide new theoretical and clinical insights into pain pathogenesis and rehabilitation.

## 2. Materials and methods

### 2.1. Patient selection study design and participants

This study was approved by the Ethics Committee of Affiliated Hospital of Hebei University of Technology. This retrospective cohort study was conducted to evaluate the efficacy of extracorporeal shockwave therapy (ESWT) in patients diagnosed with spinal-segmental herpes zoster-associated pain (ZAP) in the context of spinal musculoskeletal homeostasis (SMH) imbalance. The study included patients admitted to the between May 2023 and June 2024.

**Figure 1.**
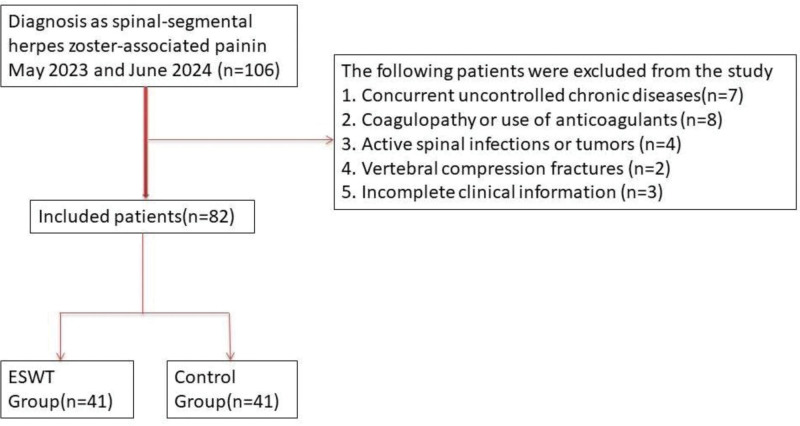
Inclusion and exclusion table for patients with zoster-associated pain. ESWT = extracorporeal shockwave therapy.

A total of 82 adult inpatients were enrolled based on clinical diagnosis and spinal MRI evidence. Patients were divided into 2 groups according to their medical records: the control group (C group, n = 41), who received standard medical treatment, and the ESWT group (E group, n = 41), who received shockwave therapy in addition to standard care. Inclusion criteria were: age between 50 and 80 years, confirmed segmental ZAP affecting thoracic or lumbar spinal dermatomes, symptom duration ≤1 month, and available musculoskeletal imaging and assessment data. Exclusion criteria included: concurrent uncontrolled chronic diseases (e.g., diabetes, heart failure), coagulopathy or use of anticoagulants, active spinal infections or tumors, vertebral compression fractures, and incomplete clinical information. This study was approved by the Affiliated Hospital of Hebei University of Technology’s Ethics Committee (Fig. 1). Because this study was retrospective, treatment allocation was not randomized. ESWT was introduced as an adjunctive therapeutic option in our institution during the study period. Patients who met the clinical criteria and had no contraindications were offered ESWT in addition to standard care. The decision to receive ESWT was influenced by several practical factors, including physician clinical judgment, patient willingness to undergo the procedure, and the availability of the ESWT device during hospitalization. Consequently, group assignment was determined retrospectively based on the treatment recorded in the medical records rather than through prospective randomization.

### 2.2. Treatment protocol

All patients received standard pharmacological management based on the Chinese expert consensus on zoster-associated pain (ZAP). This included antiviral agents (e.g., acyclovir or valacyclovir), neuropathic pain medications (such as pregabalin or amitriptyline), nonsteroidal anti-inflammatory drugs (NSAIDs), and appropriate supportive care.

In the ESWT group, patients received additional extracorporeal shockwave therapy (ESWT) targeted to the paraspinal muscles adjacent to the affected spinal segments. A Dornier electromagnetic shockwave device was used, delivering 4000 pulses/session, twice weekly for 3 consecutive weeks (6 sessions total). Treatment parameters included an energy flux density of 0.25 mJ/mm^2^, a frequency of 4 to 8 Hz, and pressure between 1 and 5 MPa. Therapy was applied under clinician supervision, with real-time adjustment based on patient tolerance and pain localization.

Both groups underwent a musculoskeletal health assessment using the Musculoskeletal Homeostasis Health Assessment (MHHA) questionnaire within 24 hours of admission, evaluating risk factors such as physical inactivity, postural habits, and previous spinal complaints. Additionally, spinal MRI was performed to assess local degenerative changes, including disc protrusion, paraspinal muscle atrophy, fatty infiltration, and ligamentous thickening. Findings from the MRI were scored using the Spinal Musculoskeletal Imaging Score (SMIS), a validated 20-item system ranging from 0 to 100, with higher scores indicating greater structural abnormality. The MHHA and SMIS scores guided individual rehabilitation advice in both groups, including recommendations on posture, mobility, and activity adjustment during hospitalization and follow-up.

### 2.3. Outcome measures and data collection

Primary outcomes were pain intensity (Numerical Rating Scale, NRS), sleep quality (Pittsburgh Sleep Quality Index, PSQI), and Spinal Musculoskeletal Imaging Score (SMIS). These were assessed at 4 time points: 48 hours after admission, 1 week posttreatment, 1 month post-discharge, and 3 months post-discharge. Secondary outcomes included the incidence of postherpetic neuralgia (PHN), defined as persistent pain beyond 90 days from onset.

All clinical data were collected retrospectively from the hospital’s electronic medical record system. Assessments were performed by independent, blinded physicians and nurses trained in pain evaluation and imaging scoring.

### 2.4. Data analysis

Descriptive statistics were used to summarize baseline demographic and clinical characteristics. Continuous variables were expressed as mean ± standard deviation and analyzed using independent-samples *t* tests. Categorical variables were compared using chi-square or Fisher exact tests. Repeated measures were assessed using ANOVA. A *P*-value < .05 was considered statistically significant. All statistical analyses were conducted using SPSS version 26.0 (IBM Corp., Armonk). Imaging scoring was validated through dual-review with interrater reliability testing.

## 3. Results

### 3.1. Baseline characteristics of patients in control and ESWT groups

A total of 82 hospitalized patients with segmental herpes zoster-associated pain (ZAP) were included in the study. Based on clinical treatment records, 41 patients received standard treatment alone (control group, C group), while the other 41 received adjunctive extracorporeal shockwave therapy (ESWT group, E group). Both groups were comparable in terms of age, sex distribution, body mass index (BMI), disease duration prior to admission, and the prevalence of comorbidities such as cardiovascular disease, diabetes, and osteoporosis-related pain (*P* > .05 for all, Table 1).

**Table 1:** Baseline information of patients with zoster-associated pain (ZAP) at admission (n = 82).

Variable	ESWT group (n = 41)	Control group (n = 41)	*P*-value*
Gender			
Male	22 (53.7)		24 (58.5)
Female	19 (46.3)	17 (41.5)	
Age			.801
≤70	28 (68.3)	30 (73.2)	
>70	13 (31.7)	11 (26.8)	
BMI (kg/m^2^)	24.8 ± 3.1	24.2 ± 3.4	.392
Spinal segment affected			.672
Thoracic (T1–T12)	32 (78.0)	33 (80.5)	
Lumbar (L1–L5)	9 (22.0)	8 (19.5)	
Side of pain			.515
Left	20 (48.8)	17 (41.5)	
Right	21 (51.2)	24 (58.5)	
ZAP duration before admission			.846
≤2 wk	25 (61.0)	24 (58.5)	
>2 wk	16 (39.0)	17 (41.5)	
Comorbidities			.472
Hypertension	14 (34.1)	16 (39.0)	
Diabetes mellitus	10 (24.4)	12 (29.3)	
Osteoporotic back pain	7 (17.1)	9 (22.0)	
Paraspinal muscle fat infiltration (MRI)	31 (75.6)	29 (70.7)	.612
Myofascial thickening	26 (63.4)	25 (61.0)	.826
Disc degeneration	19 (46.3)	17 (41.5)	.668
Foraminal stenosis	11 (26.8)	13 (31.7)	.625
Baseline SMIS (score)	54.6 ± 7.3	53.9 ± 6.8	.581
Baseline NRS (pain score)	7.1 ± 1.0	7.2 ± 1.1	.792
Baseline PSQI (sleep score)	12.3 ± 2.1	12.6 ± 2.4	.645

Values are presented as mean ± standard deviation (SD).

BMI = body mass index, ESWT = extracorporeal shockwave therapy, MRI = magnetic resonance imaging, NRS = Numerical Rating Scale, PSQI = Pittsburgh Sleep Quality Index, SD = standard deviation, SMIS = Spinal Musculoskeletal Imaging Score, ZAP = zoster-associated pain.

* *P*-values are derived from independent-samples *t* tests.

All patients underwent spinal MRI upon admission. Musculoskeletal abnormalities – such as paraspinal muscle fat infiltration, disc protrusion, and intervertebral foramen narrowing – were present in 90.2% (74/82) of the patients, with no significant differences between groups at baseline (*P* > .05).

### 3.2. Comparison of pain intensity (NRS) between groups

Pain intensity was evaluated using the Numerical Rating Scale (NRS) at 4 key time points: 48 hours after admission, 1 week after treatment initiation, and at 1 and 3 months following discharge. As detailed in Table 2, baseline NRS scores were comparable between the ESWT group (7.1 ± 1.0) and the control group (7.2 ± 1.1), with no statistically significant difference (*P* = .792). At 1 week, both groups showed a reduction in pain, but the difference remained nonsignificant (*P* = .067). However, at the 1-month mark, patients in the ESWT group reported significantly lower NRS scores (2.4 ± 0.9) than those in the control group (3.3 ± 1.1), with a statistically significant difference (*P* = .004). This difference became even more pronounced at 3 months, with the ESWT group showing near-complete resolution of pain (1.2 ± 0.6) compared to persistent discomfort in the control group (2.6 ± 1.0, *P* < .001). In terms of magnitude of pain relief, ΔNRS from baseline to 3 months was –5.9 ± 1.3 in the ESWT group versus –4.6 ± 1.5 in the control group (*P* < .001). Furthermore, 92.7% of patients in the ESWT group achieved NRS scores ≤ 3 by 3 months, compared to only 58.5% in the control group (*P* < .001), indicating a higher proportion of clinically meaningful pain relief.

**Table 2:** Comparison of pain intensity (NRS scores) and pain relief between groups at each time point.

Pain indicator	ESWT group (n = 41)	Control group (n = 41)	*P*-value†
NRS score (mean ± SD)			
48 h after admission	7.1 ± 1.0		7.2 ± 1.1
1 wk after treatment	4.9 ± 0.8	5.3 ± 1.0	.067
1 mo after discharge	2.4 ± 0.9	3.3 ± 1.1	.004*
3 mo after discharge	1.2 ± 0.6	2.6 ± 1.0	<.001**
ΔNRS (change from baseline)			
Week 1 – baseline	–2.2 ± 0.7	–1.9 ± 0.8	.084
Month 1 – baseline	–4.7 ± 1.1	–3.9 ± 1.2	.007*
Month 3 – baseline	–5.9 ± 1.3	–4.6 ± 1.5	<.001**
Patients achieving NRS ≤ 3 (n, %)‡			
At 1 mo	31 (75.6%)	18 (43.9%)	.003**
At 3 mo	38 (92.7%)	24 (58.5%)	<.001**

Values are expressed as mean ± standard deviation (SD) or number (percentage).

ESWT = extracorporeal shockwave therapy, NRS = Numerical Rating Scale, ΔNRS = change in Numerical Rating Scale from baseline, SD = standard deviation.

† *P*-values were calculated using independent-samples *t*-tests for continuous variables and chi-square tests for categorical variables.

‡ NRS ≤ 3 was considered clinically meaningful pain relief.

* *P* < .05 indicates significance.

** *P* < .01 indicates high statistical significance.

### 3.3. Comparison of sleep quality (PSQI) between groups

Sleep quality was assessed using the Pittsburgh Sleep Quality Index (PSQI), where higher scores reflect poorer sleep. As shown in Table 3, no significant difference was observed at baseline between the ESWT group (12.3 ± 2.1) and the control group (12.6 ± 2.4; *P* = .645). After 1 week of treatment, the ESWT group demonstrated a greater improvement in sleep quality with PSQI scores of 8.5 ± 1.9 versus 9.6 ± 2.0 in the control group (*P* = .018). At 1 month, the ESWT group continued to outperform the control group (5.2 ± 1.6 vs 7.3 ± 2.1, *P* < .001), with this advantage persisting at 3 months (3.6 ± 1.3 vs 6.1 ± 2.0, *P* < .001). The reduction in PSQI from baseline to 3 months (ΔPSQI) was also significantly greater in the ESWT group (–8.7 ± 2.1) compared to the control group (–6.5 ± 2.4, *P* < .001). In terms of clinical sleep recovery (defined as PSQI ≤ 5), 82.9% of ESWT patients met this threshold by 3 months, compared to only 43.9% of controls (*P* < .001), reinforcing the sustained benefit of ESWT on sleep restoration.

**Table 3:** Comparison of sleep quality (PSQI scores) and sleep recovery between groups at each time point.

Sleep indicator	ESWT group (n = 41)	Control group (n = 41)	*P*-value†
PSQI score (mean ± SD)			
48 h after admission	12.3 ± 2.1		12.6 ± 2.4
1 wk after treatment	8.5 ± 1.9	9.6 ± 2.0	.018*
1 mo after discharge	5.2 ± 1.6	7.3 ± 2.1	<.001**
3 mo after discharge	3.6 ± 1.3	6.1 ± 2.0	<.001**
ΔPSQI (change from baseline)			
Week 1 – baseline	–3.8 ± 1.2	–3.0 ± 1.4	.029*
Month 1 – baseline	–7.1 ± 1.8	–5.3 ± 2.0	<.001**
Month 3 – Baseline	–8.7 ± 2.1	–6.5 ± 2.4	<.001**
Patients achieving PSQI ≤ 5 (n, %)‡			
At 1 mo	26 (63.4%)	13 (31.7%)	.005**
At 3 mo	34 (82.9%)	18 (43.9%)	<.001**

Values are expressed as mean ± standard deviation (SD) or number (percentage).

ESWT = extracorporeal shockwave therapy, PSQI = Pittsburgh Sleep Quality Index, ΔPSQI = change in PSQI from baseline, SD = standard deviation.

† *P*-values were calculated using independent-samples *t* tests (for continuous data) or chi-square tests (for categorical comparisons).

‡ PSQI ≤ 5 was considered indicative of clinically meaningful sleep recovery.

* *P*< .05 indicates statistical significance.

** *P* < .01 indicates high statistical significance.

### 3.4. Comparison of musculoskeletal imaging scores (SMIS) between groups

Structural spinal integrity was evaluated using the Spinal Musculoskeletal Imaging Score (SMIS), a composite index derived from spinal MRI assessments. Table 4 presents SMIS scores at each time point. At baseline, both groups had similar SMIS values (54.6 ± 7.3 in the ESWT group vs 53.9 ± 6.8 in the control group; *P* = .581). After 1 week, small improvements were observed in both groups, but no significant differences emerged (*P* = .327). At 1 month, however, the ESWT group exhibited a significantly lower SMIS (45.8 ± 5.4) compared to the control group (50.3 ± 6.2; *P* = .002), and this gap widened by the 3-month follow-up (40.2 ± 5.0 vs 47.1 ± 5.9; *P* < .001). The average reduction in SMIS from baseline to 3 months (ΔSMIS) was–14.4 ± 3.9 in the ESWT group versus –6.8 ± 3.5 in the control group (*P* < .001). Furthermore, 82.9% of patients in the ESWT group achieved ≥20% structural improvement, compared to 41.5% in the control group (*P* < .001). These findings suggest that ESWT facilitates not only symptom relief but also measurable structural musculoskeletal recovery observable via imaging.

**Table 4:** Comparison of Spinal Musculoskeletal Imaging Score (SMIS) between groups at each time point.

Structural indicator	ESWT group (n = 41)	Control group (n = 41)	*P*-value†
SMIS score (mean ± SD)			
48 h after admission	54.6 ± 7.3		53.9 ± 6.8
1 wk after treatment	51.0 ± 6.1	52.2 ± 6.5	0.327
1 mo after discharge	45.8 ± 5.4	50.3 ± 6.2	.002*
3 mo after discharge	40.2 ± 5.0	47.1 ± 5.9	<.001*
ΔSMIS (change from baseline)			
Week 1 – baseline	–3.6 ± 2.5	–1.7 ± 2.4	.001*
Month 1 – baseline	–8.8 ± 3.1	–3.6 ± 2.8	<.001*
Month 3 – baseline	–14.4 ± 3.9	–6.8 ± 3.5	<.001*
Patients achieving ≥ 20% SMIS improvement (n, %)‡			
At 1 mo	21 (51.2%)	9 (22.0%)	.006*
At 3 mo	34 (82.9%)	17 (41.5%)	<.001*

Values are expressed as mean ± standard deviation (SD) or number (percentage).

ESWT = extracorporeal shockwave therapy, SD = standard deviation, SMIS = Spinal Musculoskeletal Imaging Score, ΔSMIS = change in SMIS from baseline.

† *P*-values were calculated using independent-samples *t* tests or chi-square tests as appropriate.

‡ ≥20% reduction in SMIS was considered clinically meaningful musculoskeletal structural improvement.

** *P* < .01 indicates high statistical significance.

## 4. Discussion

Zoster-associated pain (ZAP) remains a common and disabling complication of herpes zoster, particularly in elderly individuals, often leading to long-term neuropathic pain and reduced quality of life.^[2,3,13,14]^ Although antiviral and neuropathic pain therapies form the cornerstone of management, their effectiveness remains limited, especially in preventing postherpetic neuralgia (PHN). Our study assessed the impact of extracorporeal shockwave therapy (ESWT), a noninvasive neuromodulatory technique, in patients with spinal-segmental ZAP characterized by musculoskeletal homeostasis (SMH) imbalance.^[9,15-17]^ The results suggest that ESWT not only improves pain and sleep quality but also enhances musculoskeletal structural recovery and reduces the incidence of PHN.

The therapeutic benefits of ESWT observed in our study are consistent with prior research on its use in musculoskeletal conditions and chronic pain syndromes.^[15,17]^ Notably, patients who received ESWT exhibited greater improvements in pain intensity, as measured by NRS, and reported significantly better sleep quality (PSQI) as early as 1 week after treatment initiation. These findings highlight the potential of ESWT to modulate peripheral and central pain processing mechanisms in ZAP. Moreover, the reduction in Spinal Musculoskeletal Imaging Scores (SMIS) over time suggests that ESWT may promote tissue-level structural remodeling, potentially alleviating local biomechanical compression or inflammatory sensitization of nerve roots. Importantly, the incidence of PHN in the ESWT group was markedly lower compared to the control group (4.9% vs 31.7%, *P* < .01), underscoring the potential role of ESWT in not only symptom management but also secondary prevention of chronic neuropathic pain. This supports emerging evidence that early physical interventions targeting paraspinal muscles and fascia can influence the trajectory of nerve inflammation and neuroplasticity. Our study adds to this growing body of evidence by incorporating a structural perspective through musculoskeletal imaging analysis and providing a quantitative framework for evaluating spinal muscle-fascial dysfunction in the context of ZAP.

Another key finding of our study is the significant improvement in SMIS among patients receiving ESWT. The restoration of spinal musculoskeletal homeostasis may be a critical factor in sustaining long-term pain relief and functional recovery. By reducing localized muscle tension, improving vascular perfusion, and decreasing nociceptive input, ESWT may support a more favorable healing environment for inflamed or demyelinated nerve roots. This aligns with previous findings in the literature that mechanical dysfunction and postural instability may perpetuate neuropathic pain syndromes if not concurrently addressed with neural treatment.^[17-19]^

Despite these encouraging findings, several limitations should be acknowledged. First, this study was retrospective and non-randomized, which introduces the possibility of selection bias and confounding by indication. ESWT was offered as an adjunctive therapy during the study period, and the decision to receive this intervention was influenced by physician judgment, patient willingness, and equipment availability. As a result, patients who received ESWT may have differed from those receiving standard treatment in ways not fully captured in the available clinical data, such as treatment motivation or adherence to rehabilitation recommendations. These factors could potentially influence the observed effect size and may partially exaggerate the apparent benefits of ESWT.

Second, this was a single-center study with a relatively small sample size, which may limit the generalizability of the findings. Third, although spinal MRI and SMIS were used to quantify musculoskeletal dysfunction, additional techniques such as diffusion tensor imaging or electromyography could provide more detailed insights into neuromuscular changes.

Finally, the follow-up duration was limited to 3 months. While PHN is commonly defined as pain persisting beyond 3 months after rash onset, some patients may develop or continue to experience PHN beyond this time point. Therefore, the long-term preventive effect of ESWT on PHN requires further investigation through prospective studies with longer follow-up periods. Future randomized controlled trials with larger sample sizes and longer follow-up periods are required to confirm the magnitude of the therapeutic effect and to minimize potential selection bias.

## 5. Conclusion

In conclusion, this study demonstrates that extracorporeal shockwave therapy (ESWT), when applied to patients with spinal-segmental ZAP and musculoskeletal imbalance, significantly improves clinical outcomes. ESWT not only accelerates pain relief and sleep restoration but also enhances musculoskeletal structural recovery and reduces the incidence of postherpetic neuralgia. The integration of musculoskeletal homeostasis evaluation into the clinical management of ZAP offers a novel and promising therapeutic framework. These findings support the adoption of ESWT as an adjunct to standard treatment protocols and advocate for a multidisciplinary approach to managing ZAP that considers both neurological and musculoskeletal components. Further prospective and large-scale studies are warranted to establish standardized protocols and to explore the long-term benefits and mechanisms of ESWT in this population.

## Acknowledgments

Thanks to the nurses in the department for their help with the project.

## Author contributions

**Conceptualization:** Fang Cheng, Yanzhong Yu, Jing Zhang.

**Data curation:** Fang Cheng, Yanzhong Yu, Jing Zhang.

**Formal analysis:** Fang Cheng, Yanzhong Yu, Jing Zhang.

**Funding acquisition:** Fang Cheng.

**Investigation:** Fang Cheng.

**Writing – original draft:** Fang Cheng.

**Writing – review & editing:** Fang Cheng.

## Correction

This article was originally published with an incorrect affiliation institution. This has now been corrected online from “Affiliated Hospital of Hebei University of Technology” to “The Affiliated Hospital of Hebei University of Engineering.”
